# Block Copolymer Based Porous Carbon Fiber—Synthesis, Processing, and Applications

**DOI:** 10.1021/accountsmr.4c00404

**Published:** 2025-02-02

**Authors:** Adeel Zia, Yue Zhang, Akshara Paras Parekh, Guoliang Liu

**Affiliations:** † Department of Chemistry, Virginia Tech, Blacksburg, Virginia 24061, United States; ‡ Department of Chemical Engineering, Department of Material Science and Engineering, Macromolecules Innovation Institute, Virginia Tech, Blacksburg, Virginia 24061, United States

## Abstract

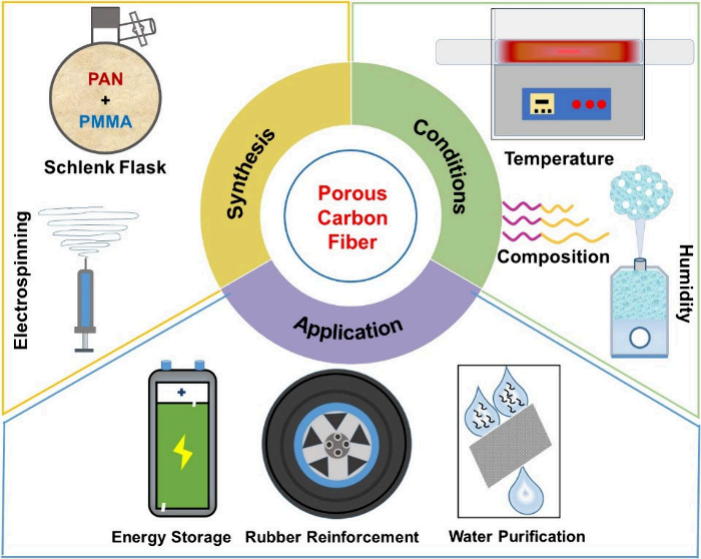

Carbon is an abundant material with remarkable thermal, mechanical, physical, and chemical properties. Each allotrope has unique structures, properties, functionalities, and corresponding applications. Over the past few decades, various types of carbon materials such as graphene, carbon nanotubes, carbon quantum dots, and carbon fibers have been produced, finding applications in energy conversion and storage, water treatment, sensing, polymer composites, and biomedical fields. Among these carbon materials, porous carbons are highly interesting owing to their large surface areas and massive active sites to interact with molecules, ions, and other chemical species. The pore size and pore size distributions can be tunable (micro-, meso-, and macro-pores), providing chemical species with hierarchical structures to transport with low resistances. In this context, designing carbon precursors and preparing porous carbon with desired structures, properties, and functionalities are highly significant.

Polymers are versatile carbon precursors. Designing the polymer precursors that facilitate the formation of well-controlled pores is an effective strategy to prepare porous carbons. In particular, porous carbon fibers (PCFs) in a fibrous format offer additional features of hierarchical porosity control, increased surface area, and fast ion transport. The most common approach to synthesizing PCFs is to use sacrificial agents (e.g., homopolymers of polystyrene (PS) and poly(methyl methacrylate) (PMMA), inorganic nanoparticles, and other additives) in a matrix of polyacrylonitrile (PAN) as the carbon fiber precursor. However, the nonuniform mixing of sacrificial agents in the PAN matrix results in PCFs with nonuniform pores and wide pore size distributions. Moreover, complete removal of the inorganic additives is challenging and sometimes requires the use of hazardous chemicals. Therefore, developing innovative methods for synthesizing PCFs is imperative to advance these engineering materials for emerging applications.

In this Account, we summarize our efforts on the use of block copolymer precursors to prepare PCFs with tunable pore sizes and pore size distributions for a series of applications. First, we will introduce the synthesis methodologies for preparing PCFs. We have used reversible addition–fragmentation chain transfer (RAFT) polymerization to synthesize block copolymer precursors. Second, we will discuss the effects of preparation conditions on the properties of PCFs. The mechanical and electrical properties highly depend on the composition of the block copolymer, pyrolysis conditions, and humidity level during the fiber spinning process. Lastly, we will discuss the effects of controlled porosity on the surface area, electrical/ionic conductivity, and polymer-matrix interactions, which are crucial for applications including energy storage (e.g., batteries and supercapacitors), fiber-reinforced polymer composites, separation, and filtration.

## 1 Introduction

Carbon fibers are one-dimensional materials that have superb mechanical strength and are used as fillers in polymers to create reinforced composites.^1,2^ By introducing rich functionalities on the surfaces, carbon fibers can interact with ions, molecules, and other species, representing an innovative material for emerging applications such as electrochemical energy storage and water purification.^3−7^ Conventional carbon fibers are often derived from pitch,^5,8^ biofeedstock (cellulose, lignin),^9^ polyacrylonitrile (PAN),^10^ and commodity polyolefins.^11^These traditional fibers are typically solid carbon filaments with smooth surfaces, limiting the amount of surface area for high-density energy storage and ion/molecule interactions.^12−14^ Introducing porosity is an excellent way to enhance the surface area for emerging applications.^15−18^

**Figure 1:**
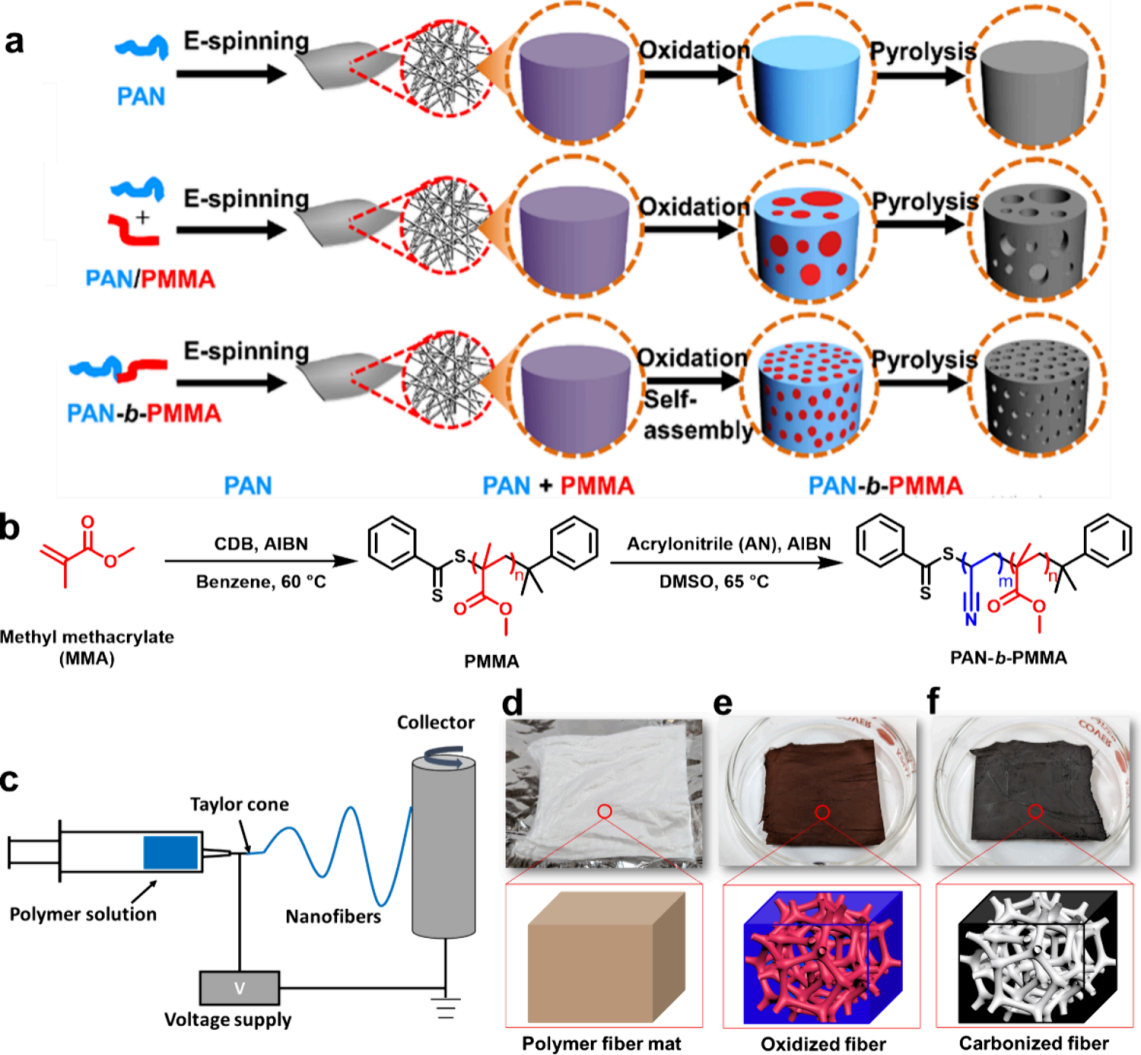
(a) Different methods for synthesizing nonporous and porous carbon fibers. Reproduced with permission from ref (3). Copyright 2019 The Authors. (b) Synthesis route of PAN-*b*-PMMA via RAFT polymerization using cumyl dithiobenzoate (CDB), azobis(isobutyronitrile) (AIBN), acrylonitrile (AN), and dimethyl sulfoxide (DMSO). (c) Schematic of electrospinning of polymer precursors into fibers. (d) After electrospinning, PAN-*b*-PMMA block copolymer fiber mats. (e) After oxidation, PAN-*b*-PMMA microphase separates into interconnected PMMA nanodomains (red) in a PAN matrix (blue). (f) After pyrolysis, PMMA is removed to generate mesopores and PAN matrix is converted to carbon, resulting in an interconnected porous structure (gray) in a carbon matrix (black). Reproduced with permission from ref (21). Copyright 2020 The Royal Society of Chemistry.

In 2019, our laboratory established a method of producing PCFs from a block copolymer precursor of poly(acrylonitrile-*block*-methyl methacrylate) (PAN-*b*-PMMA). In the designed block copolymer, PAN is the carbon source. The cyclization reaction during oxidation forms a thermally stable ladder-like structure, which can give a high carbon yield after pyrolysis. PMMA is a sacrificial block and fully decomposes after pyrolysis, forming a porous structure. The PCFs show uniform porosity, high surface area, good electrical conductivity, and excellent ion transport properties. Importantly, the copolymerization of PAN with a sacrificial polymer block (e.g., poly(methyl methacrylate) or PMMA) ensures the nanoscale “mixing” of the immiscible polymers, and yet the microphase separation of the two polymers drives the formation of nanoscale polymer domains to produce highly uniform porous structures (Figure 1a).^3,19^ The block copolymer-derived PCFs possess an interconnected network of micro-, meso-, and macro-pores, which are important for various applications, including energy storage, separation, filtration, and polymer composites. Since then, we have conducted systematic studies to investigate the conditions for preparing PCFs with enhanced properties, i.e., controlled porosity, improved mechanical properties, flexibility, and electrochemical performance.^3,4,20−26^

To provide future research directions of porous carbons in general, in a recent perspective we briefly summarized the synthesis of PCFs from block copolymers and shared our opinion of this particular type of porous carbon for applications such as energy storage and water treatment.^27^ To provide clear guidance for future research in the areas of PCFs, this Account aims to comprehensively detail and thoroughly analyze the synthesis conditions, morphologies, and properties of PCFs. We particularly focus on the influence of various synthesis conditions on the morphology and properties of PCFs. First, we will discuss the synthesis of PCFs, including steps of block copolymer precursor synthesis, electrospinning, oxidation, and pyrolysis. Then, we will discuss the effects of precursor composition and preparation conditions on the physical properties of PCFs. Next, we will summarize the applications of PCFs in energy storage (e.g., supercapacitors and batteries), water purification (e.g., organic dye removal and water desalination), and fillers for natural rubber. We hope this Account will help the readers understand the mechanisms and conditions for synthesizing PCFs to achieve desired properties and functionalities, thus inspiring the development of robust PCFs to meet future technological demands.

## 2 Synthesis of PCFs

### 2.1 Synthesis of Polymer Precursors

RAFT polymerization was employed to synthesize PAN-*b*-PMMA (Figure 1b). The process was initiated by dissolving a mixture of methyl methacrylate (MMA), a chain transfer agent cumyl dithiobenzoate (CDB), and an initiator azobis(isobutyronitrile) (AIBN), in benzene within a Schlenk flask. The solution underwent three rounds of freeze–pump–thaw and nitrogen backfilling to eliminate any residual oxygen. After that, the solution was subjected to heat in an oil bath at 60 °C for various durations of time, resulting in the synthesis of PMMA macro-chain transfer agents (PMMA macro-CTAs) with varying number-average molecular weights (*M*_n_). The resulting PMMA was precipitated in methanol, filtered, and dried. To synthesize PAN-*b*-PMMA, a mixture of PMMA macro-CTA, AIBN, and acrylonitrile (AN) was dissolved in dimethyl sulfoxide (DMSO) in a Schlenk flask, followed by degassing through freeze–pump–thaw rounds. The reaction mixture was then placed in an oil bath at 65 °C under a nitrogen atmosphere. The resulting PAN-*b*-PMMA was precipitated, purified, and subjected to vacuum drying.

### 2.2 Fabrication of Polymer Fiber Precursors

We employed electrospinning to fabricate block copolymer fiber mats (Figure 1c). Typically, PAN-*b*-PMMA was dissolved in dimethylformamide (DMF) (16 wt %) at 65 °C for 4 h, following the electrospinning at a rate of 1 mL h^–1^, and a voltage of 15–20 kV. A white polymer fiber mat was collected in an in-house-built rotary drum. The electrospun polymer fiber mats were composed of randomly oriented nanoscale fibers. These fibers scattered visible light in all directions, giving fiber mats a white appearance. The color of the polymer (PAN-*b*-PMMA) used for electrospinning was either light yellow or white, further enhancing the visible light scattering. The polymer fiber mat exhibited high flexibility and rough surfaces due to rapid solvent evaporation during electrospinning. The polymer fiber mat after electrospinning, oxidation, and pyrolysis is shown in Figure 1d–f. Scanning electron microscopy (SEM) can reveal the detailed structures of individual fibers after electrospinning. The average diameter of our polymer fibers was 911 ± 122 nm. However, the morphology and average diameter of the polymer fiber can be tuned by the processing conditions such as solution concentration, applied voltage, feeding rate, and molecular weight.^3,4^ Higher solution concentrations have low surface tension and less solvent resulting in fibers with larger diameters. Lower voltage produces fiber with fewer bead defects. A higher feeding rate and high molecular weight of the polymer provide higher stability to the solution jet. High molecular weight has high chain entanglements providing sufficiently high viscosities resulting in a continuous solution jet that produces uniform fibers.^28,29^

### 2.3 Oxidation and Pyrolysis

To convert the block copolymer fibers into PCFs, the polymer fiber mat was initially oxidized by heating from room temperature to 280 °C at a ramp rate of 1 °C min^–1^ in the air and maintained at 280 °C for 10 h. The oxidation process results in stabilization of the PAN block due to cross-linking and cyclization reactions.^30^ The diameter of the fiber mat remained largely unchanged after oxidation. After oxidation, the fiber was subjected to pyrolysis under an inert environment (e.g., N_2_) by heating from room temperature to a high temperature (e.g., 800 °C) with a ramp rate at 10 °C min^–1^. After constant heating for 1 h, the polymer fibers resulted in PCFs with a continuous porous network.

**Figure 2:**
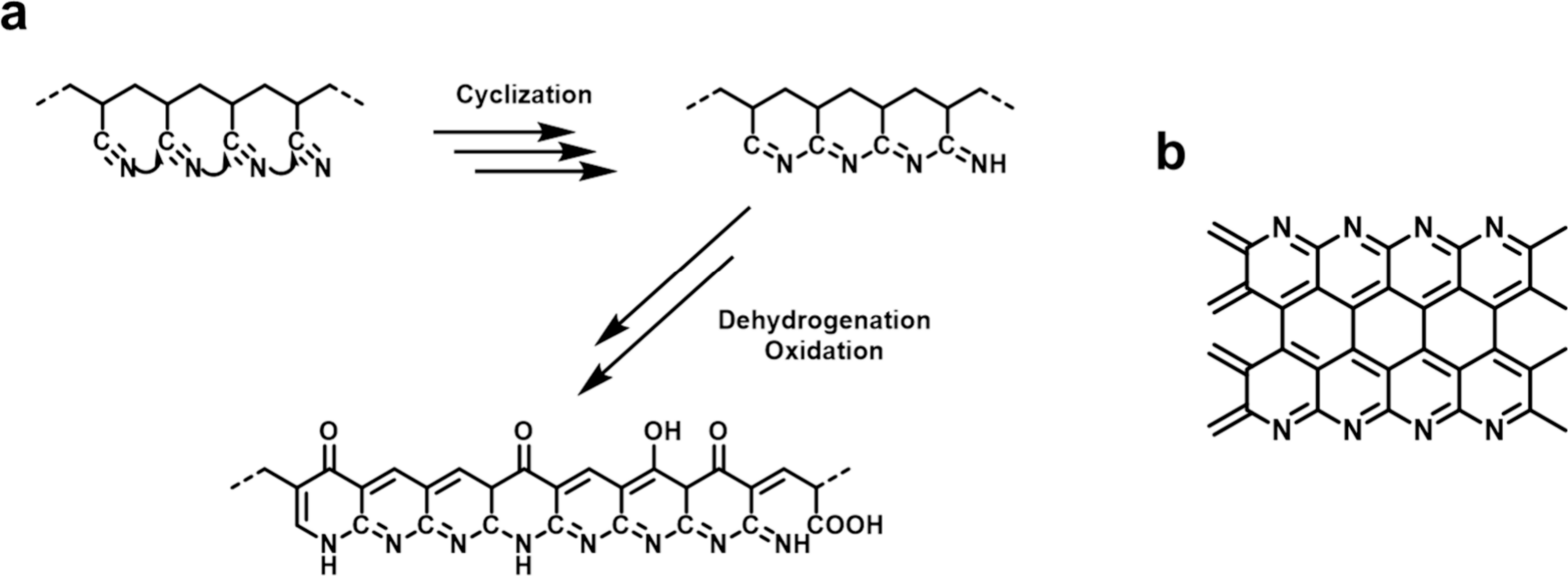
(a) Reaction pathways of PAN during oxidation. (b) Structure of N-doped carbonized PAN, which can undergo high-temperature heating to increase the graphitic level.

**Figure 3:**
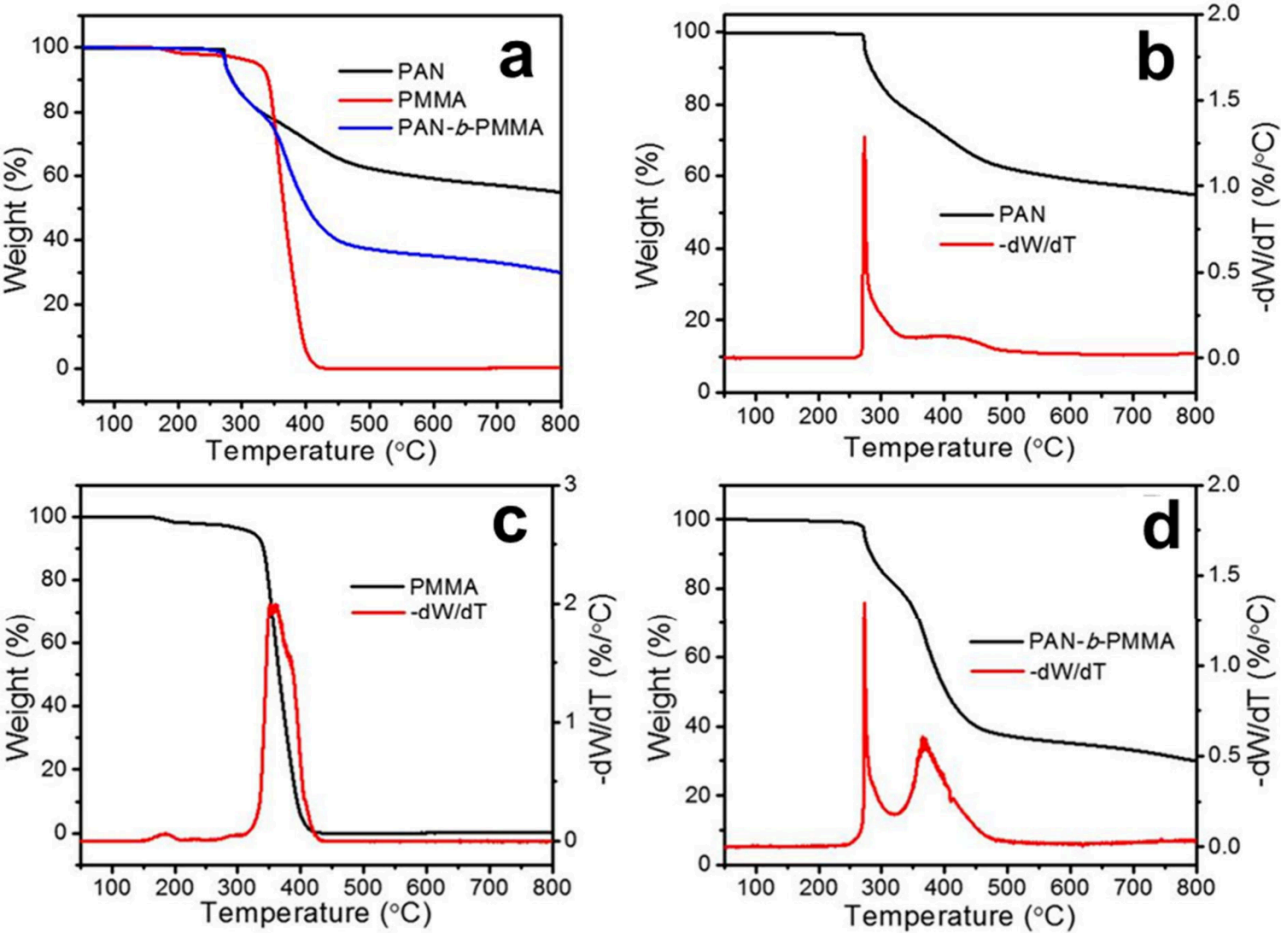
TGA profiles (a) and first derivative (−d*W*/d*T*) of the weight losses of (b) PAN, (c) PMMA, and (d) PAN-*b*-PMMA.

**Figure 4:**
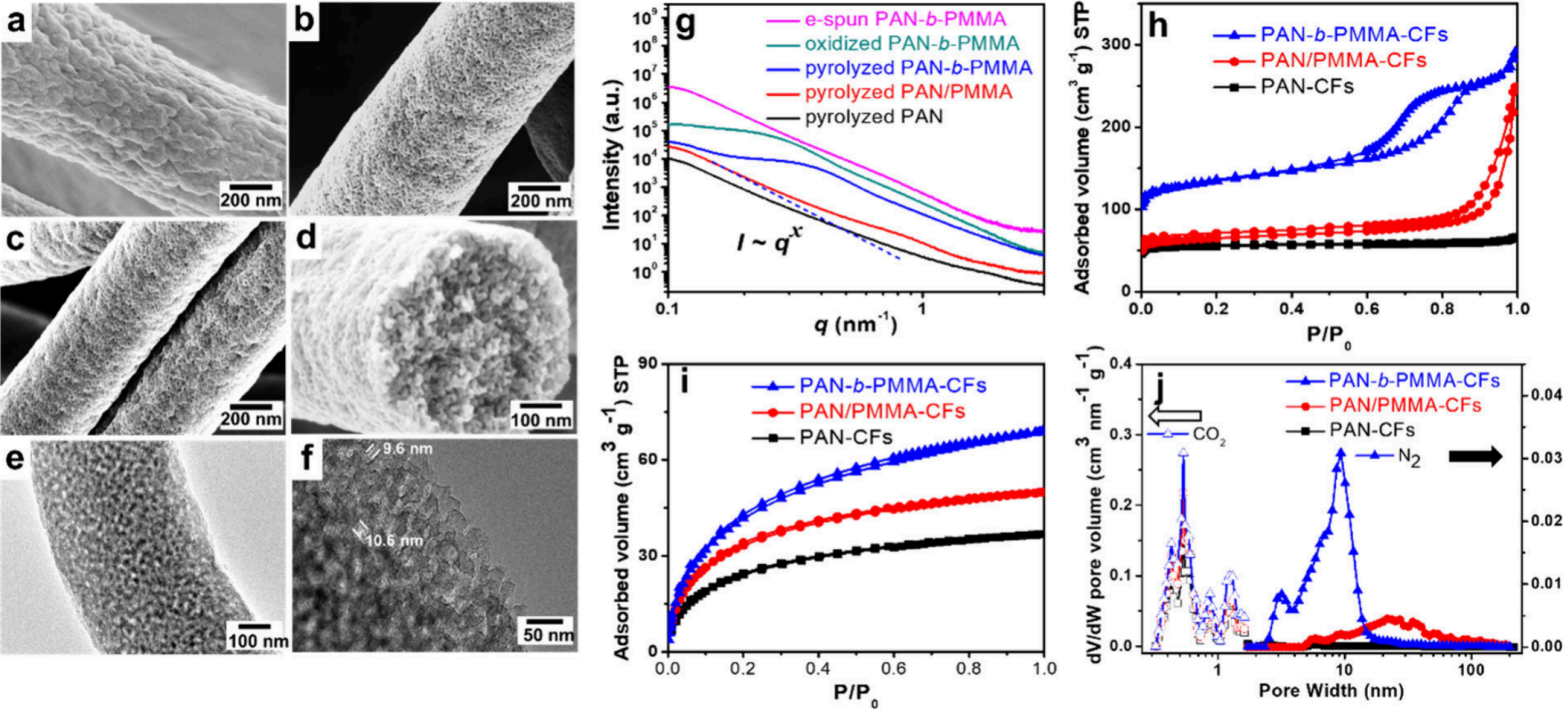
SEM images of (a) PAN-*b*-PMMA after electrospinning, (b) after oxidation at 280 °C in air, (c, d) after pyrolysis at 800 °C in N_2_. (e, f) TEM images of PCFs. (g) SAXS, and (h–j) BET analysis of CFs derived from PAN-*b*-PMMA, PAN/PMMA and PAN. Reproduced with permission from ref (3). Copyright 2019 The Authors.

Upon thermal annealing, PAN-*b*-PMMA undergoes microphase separation due to incompatibility between the two covalently bonded polymer blocks. After the microphase separation, the morphology, in principle, is governed by the Flory–Huggin interaction parameters, the molar mass of the block copolymer, and the volume ratio of the two polymer blocks. As predicted by the classical block copolymer phase diagram, the morphologies include spheres, cylinders, lamellae, and gyroid^31−33^ when PAN-*b*-PMMA is annealed in an inert atmosphere. The thermal annealing of PAN-*b*-PMMA, however, requires the presence of oxygen to induce PAN cross-linking and to ensure a high carbon yield after pyrolysis. During thermal annealing in the air, PMMA partially degrades due to oxidation, resulting in a slight weight loss in thermogravimetric analysis (TGA) (Figure 3). Meanwhile, PAN cross-links and cyclizes. The mechanism of the cross-linking and cyclization of PAN during oxidation is shown in Figure 2. In general, the linear PAN is subject to dehydrogenation and cyclization, forming a cyclized, ladder-like polymer containing nitrogen. It can be further oxidized to include some oxygen-containing functional groups. The cyclized structure is more thermally stable than linear PAN. The domains of the PAN and PMMA blocks after oxidation are easily distinguishable in SEM images (Figure 4b).

During pyrolysis, PMMA fully decomposes, as suggested by TGA (Figure 3), generating pores in the final structure. Cyclized PAN forms a turbostratic carbon structure (Figure 2), releasing gases such as HCN and N_2_. The block copolymer phase diagram applies to block copolymers without cross-linking, whose chains are subjected to interactions like van der Waals forces to form a thermodynamically equilibrated structure. However, PAN is subject to additional chemical cross-linking reaction upon heating. After the cross-linking, the molecular structure changes, and thus, the classical block copolymer phase diagram becomes inapplicable to PAN-*b*-PMMA. The block copolymer forms a bicontinuous structure after oxidation and an interconnected porous structure after pyrolysis, regardless of molecular weight and volume ratio of the polymer blocks in PAN-*b*-PMMA.^27^ This favors the formation of PCFs with continuous porous structure and high surface areas.

The carbon yield depended on the PAN volume fraction and, typically, a block copolymer with 64 vol % of PAN produced ∼30.5% carbon after pyrolysis. The resulting PCF structure remained intact, and the fiber diameters depended on the block copolymer fiber diameters (Figure 4c, d). PCFs could have a rich nitrogen content (e.g., 12.8% after pyrolysis at 800 °C), inherited from the PAN block.^34^ Notably, the substantially larger surface area (>500 m^2^/g) of PCFs than that of PAN-derived carbon fibers signified the high porosity and low density of the former.^3^ Thorough analysis using high magnification SEM, transmission electron microscopy (TEM), CO_2_ and N_2_ adsorption–desorption confirmed that PCFs possessed interconnected mesopores of approximately 10 nm in size with a total porosity of approximately 50.6% throughout the structure (Figure 4a–j).

Pretreatment such as solvent annealing can change the morphology of block copolymer and the final carbon fibers. We have not studied the effects on carbon fiber yet, but we have studied the effect of solvent annealing on mesoporous carbon thin film.^35^ The polymer film annealed in DMF formed distinguishable microphase-separated nanostructures and yielded carbon thin film with large pore size and center-to-center pore spacing, likely due to the thin-film confinement effect.^36^ The polymer films annealed in DMSO, toluene, and chloroform have less ordered structures and formed pore size similar to the carbon thin film without solvent annealing.

## 3 Effect of Preparation Conditions on PCF Properties

### 3.1 Effect of Precursor Composition

**Figure 5:**
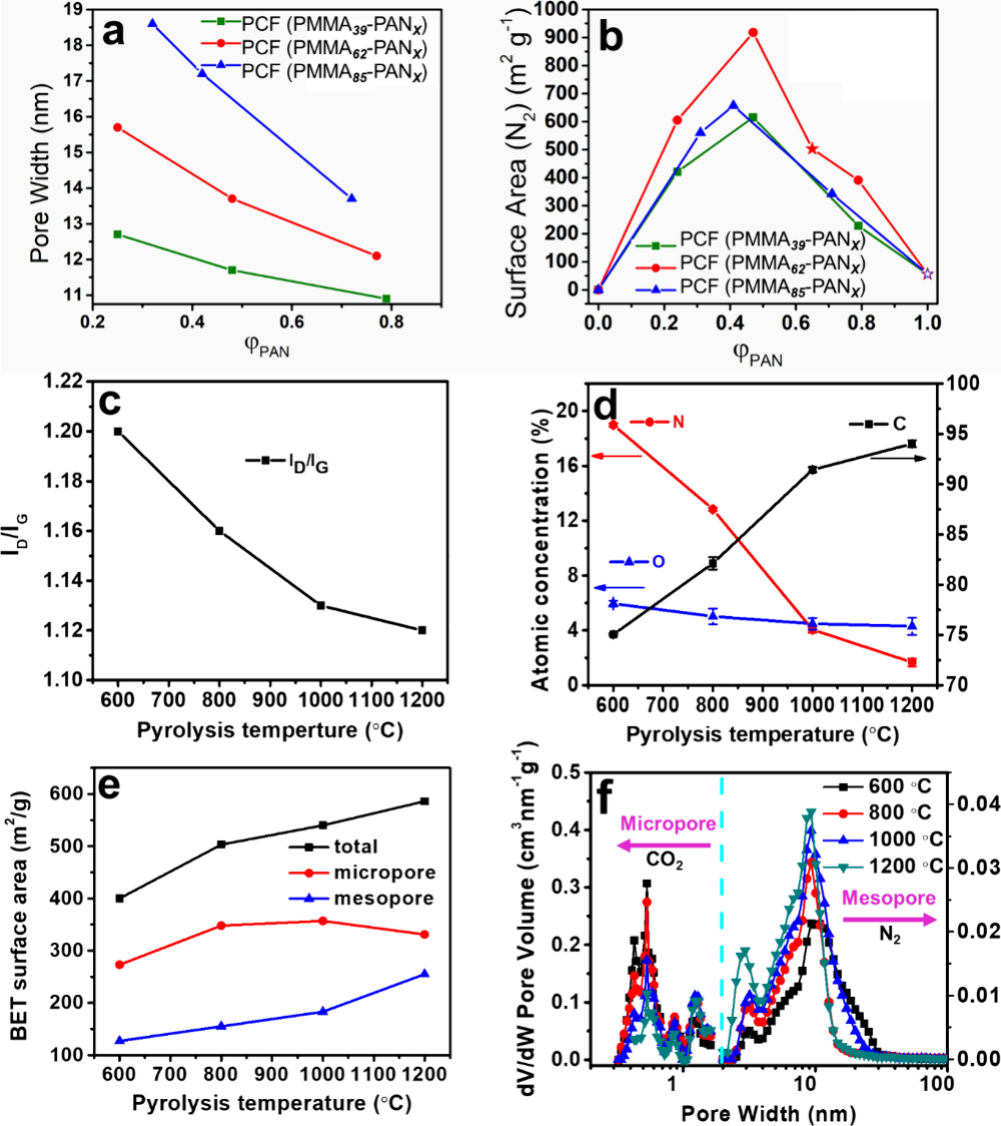
(a) Pore width and (b) surface area of PCFs from BCPs with different PAN volume fractions (ϕ_PAN_). Reproduced with permission from ref (4). Copyright 2019 American Chemical Society. (c) *I*_D_/*I*_G_ ratio, (d) atomic concentration of C, O, and N, (e) surface area and total pore volume of PCFs from different pyrolysis temperatures. (f) Micropore and mesopore size distribution calculated from CO_2_ and N_2_ isotherms. Reproduced with permission from ref (21). Copyright 2020 The Royal Society of Chemistry.

Typically, the microphase separation of PAN-*b*-PMMA forms bicontinuous structures during the oxidation process. The subsequent pyrolysis decomposes PMMA and carbonizes PAN to form PCFs. Changing the molecular weight of PMMA and PAN changes the domain size, thus altering the surface area and porous structures in PCFs. To study the effect of polymer composition, nine block copolymers with different molecular weights (from 80 kDa to 300 kDa) and different PAN volume fractions (the volume of PAN divided by that of PAN-*b*-PMMA, ϕ_PAN_ ∼ 25%, 50%, and 75%) were synthesized and yielded PCFs.^4^ The pore morphology and surface area were characterized by N_2_ adsorption and desorption. All PCFs adopted hierarchical porous structures and uniform pore size distribution due to the block copolymer microphase separation. The pore width was governed by the PMMA molecular weight and ϕ_PAN_ (Figure 5a). With the same ϕ_PAN_, block copolymers with higher PMMA molecular weight generated larger pores in PCFs because larger PMMA blocks formed larger PMMA domains during microphase separation. For block copolymers with the same PMMA molecular weight, PCFs with lower ϕ_PAN_ generated larger pores.

Despite similar PMMA domain size, lower PAN molecular weight slightly decreased the amount of carbon and increased the pore size. In each series of block copolymers with the same PMMA molecular weight, block copolymers with ϕ_PAN_ ∼ 50% generated PCFs with the highest surface areas (Figure 5b).

### 3.2 Effect of Pyrolysis Temperature

The pyrolysis of PAN-*b*-PMMA decomposes the PMMA block and carbonizes the PAN block to form porous structures in carbon fibers. Pyrolysis temperature is a critical parameter that governs the properties of PCFs. PCFs were pyrolyzed at different temperatures (600–1200 °C), and the graphitic level, heteroatom content, and porosity were characterized.^21^ The D-band (∼1310 to 1350 cm^–1^) and G-band (∼1560 to 1600 cm^–1^) in Raman spectroscopy correspond to the disordered domains and ordered graphitic structures in PCFs. The ratio of the peak intensity (*I*_D_/*I*_G_) indicated the graphitic level of PCFs, which decreased with the pyrolysis temperature. More ordered graphitic structures were formed at higher pyrolysis temperatures (Figure 5c). The increasing pyrolysis temperature reduced the nitrogen and oxygen heteroatom concentrations by releasing volatile gases such as N_2_, H_2_O, CO_2_, and NH_3_ (Figure 5d). Increasing pyrolysis temperature also increased the surface area (Figure 5e). As the pyrolysis temperature increased to 800 °C, the mesopore size slightly increased, but the mesopore size did not change drastically with the further increase. The pyrolysis temperature had an unnoticeable effect on the micropore size (Figure 5f).

### 3.3 Effect of Humidity

**Figure 6:**
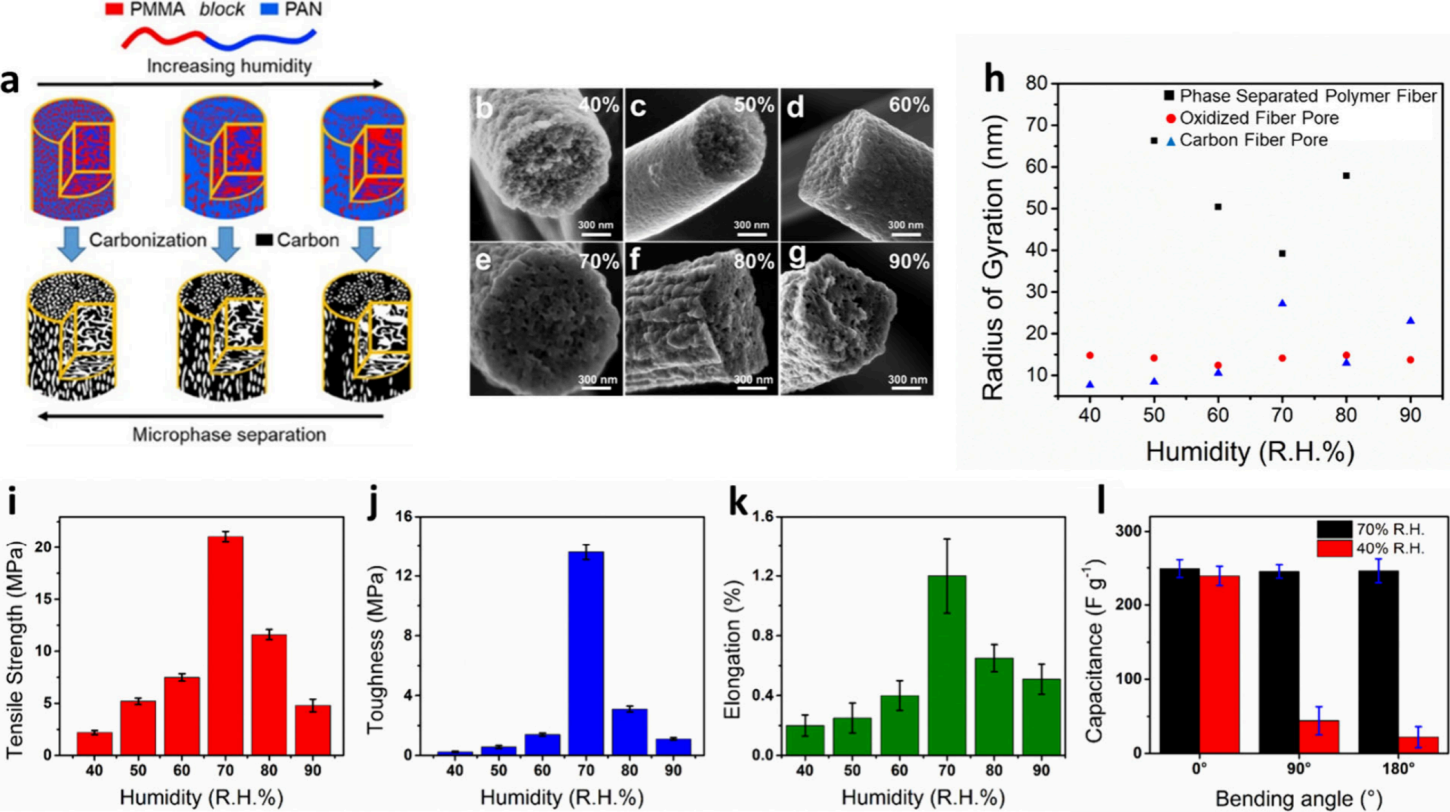
(a) With increasing humidity, the polymer fiber morphology evolved from microphase separation to vapor-induced phase separation (VIPS) and to vapor-induced precipitation. (b–g) SEM images of PCFs at different relative humidity levels from 40% to 90%. (h) Radius of gyration of PCFs at different relative humidity levels. (i) Tensile strength, (j) toughness, and (k) elongation of PCFs prepared at different humidity levels. (l) Capacitance of PCFs prepared at R.H. = 40% and 70% with different bending angles of 0°, 90°, and 180°. Reproduced with permission from ref (20). Copyright 2022 American Chemical Society.

Block copolymer-based PCFs prepared under humid conditions exhibited flexibility while maintaining high energy density and mechanical strength. PAN-*b*-PMMA fibers were electrospun under relative humidity (R.H.) of 40–90% to study the morphology change of electrospun polymer fibers and the resulting PCFs by the influence of relative humidity.^20^ Water vapor acted as a nonsolvent and affected phase separation during electrospinning. As the R.H. was increased, the polymer fiber morphology evolved from microphase separation to vapor-induced separation (VIPS) to vapor-induced precipitation (Figure 6a). At low R.H. (40–50%), the polymer fiber showed well-dispersed domains from block copolymer microphase separation, and the resulting PCFs developed uniform pores (Figure 6b, c). At midrange R.H. (60–70%), the polymer fiber reorganized and developed a PAN sheath due to the combination of microphase separation and VIPS. The resulting PCFs developed nonuniform pores and a sheath layer on the surface (Figure 6d, e). At high R.H. (80–90%), PAN sheath also formed in the polymer fiber, but larger pores appeared. The resulting PCFs showed ridge-like carbon structure and nonuniform pores in the cross-section of fibers (Figure 6f, g). The radius of gyration was extracted by the fitting of the profiles of ultrasmall-angle X-ray scattering (USAXS)/small-angle X-ray scattering (SAXS) to the United Fit (Figure 6h). The radius of gyration of phase-separated domains in polymer fibers decreased from 70 to 40 nm with an increase in R.H. from 50% to 70%. The radius of gyration increased to 60 nm at R.H. = 80%. The radius of gyration of oxidized fibers is independent of R.H. with a value around 14 nm. The increasing humidity also increased the radius of gyration of carbon fibers, with the highest value of 27.2 nm at R.H. = 70%. The humidity also altered the mechanical properties of PCFs (Figure 6i, j, k). The tensile strength, toughness, and elongation increased with the increase in R.H. from 40 to 70%, then decreased when R.H. was further increased to 90%. When the relative humidity increased to 60% and 70%, a continuous layer of carbon sheath was grown on the PCFs. This layer of carbon sheath increased the mechanical strength of PCFs. As the relative humidity further increased to 80% and 90%, beads and clumps formed on carbon fiber and developed ridge-like carbon structures, decreasing the mechanical strength of PCFs.

The PCFs prepared at R.H. = 70% had a tensile strength of 20 MPa, a toughness of 14 MPa, and an elongation of ∼1.2%. These PCFs showed flexibility while maintaining good electrochemical properties. Both PCFs prepared at R.H. = 40% and 70% were fabricated into electrodes for supercapacitors, and their capacitances were measured (Figure 6l). Both PCF electrodes showed similar capacitance (239 ± 12 and 249 ± 13 F g^–1^) when the electrodes were not bent. After the electrodes were bent 90° or 180°, the capacitance of PCF electrodes (R.H. = 40%) dropped by ∼82%, while the capacitance of PCF electrodes (R. H. = 70%) was still maintained at 249 ± 20 F g^–1^. During electrospinning at R.H. 70%, water vapor served as a nonsolvent, inducing microphase separation and VIPS. The core–sheath structure was formed by the reorganization of PMMA and PAN block by the effect of water vapor. The resulting PCFs developed a carbon sheath and improved the mechanical properties. The fabricated electrodes showed both flexibility and electrochemical performance, a potential material for flexible electronics.

## 4 Applications of PCFs

### 4.1 Energy Storage

#### 4.1.1 Supercapacitors

**Figure 7:**
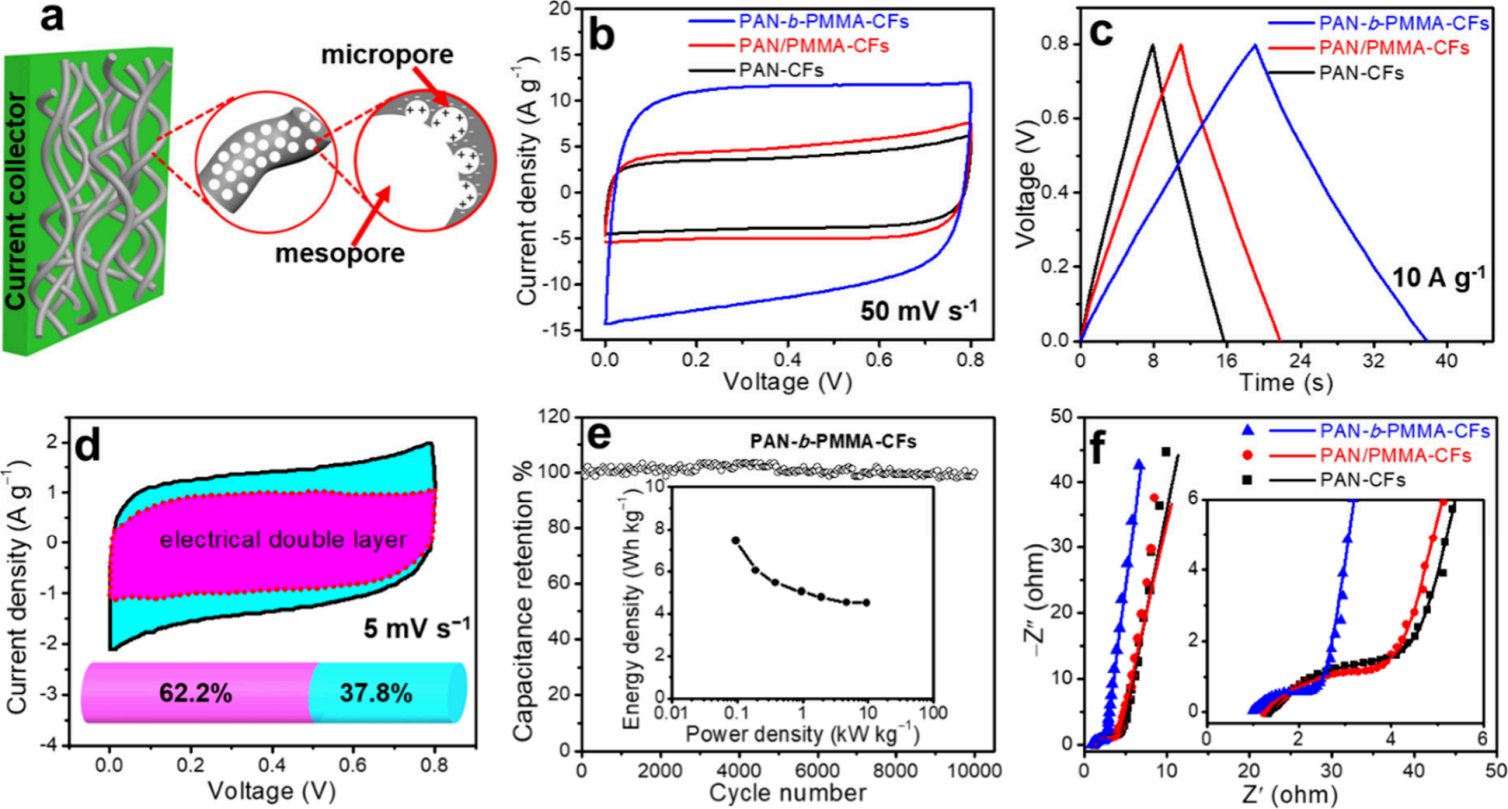
(a) Schematic of PCFs-based electrode, free from binders and conductive additives, (b) CV curves at 50 mVs^–1^ scan rate, (c) CP curves at 10 A g^–1^ current density, and (d) capacitance contributions of PAN-*b*-PMMA-CFs via Dunn method. Shaded regions show electrical double-layer capacitive (magenta) and pseudocapacitive (cyan). (e) PAN-*b*-PMMA-CFs exhibit cycling stability at a current density of 100 Ag^1–^. Ragone plot of PCFs (inset). (f) Nyquist plots of PCF with 10 mV across-current perturbation, covering a frequency range from 100 kHz to 0.1 Hz. Reproduced with permission from ref (3). Copyright 2019 The Authors.

Supercapacitors can effectively store energy and deliver it with a high power density, long cycle life, and significant current density in a short period. CFs possess extensive surface areas and abundant functionalities for engaging with ions, molecules, and other species, and thus, they are potential supercapacitor electrode materials. However, the lack of controlled porosity limits their capacity and energy density. Our group has prepared hierarchical block copolymer-derived PCFs with precisely controlled mesopores (∼10 nm) and micropores (∼0.5 nm).^3^ The electrochemical performance of these block copolymer-derived PCFs as capacitor electrodes, without any conductive additives or polymer binders, have been tested in aqueous electrolytes (e.g., 6 M KOH) (Figure 7). The PCF electrodes from PAN-*b*-PMMA showed a nearly rectangular curve in cyclic voltammograms (CV) without redox peaks even when scanned at a rate of 100 mV s^–1^, indicating a near-ideal capacitive behavior (Figure 7b). Compared to CFs prepared from polymer blends and PAN (denoted PAN/PMMA-CFs and PAN-CFs, respectively), PCFs from the block copolymer (PAN-*b*-PMMA-CFs) exhibited the maximum area captured by CV, indicating the greatest capacitance with a scan rate of 50 mV s^–1^. Chronopotentiometry (CP) and CV were used to measure gravimetric capacitances. Specifically, at 10 A g^–1^, which is a high current density, the PAN-*b*-PMMA-CFs achieved gravimetric capacitance of 226 ± 6 F g^–1^, which is more than twice as compared to PAN/PMMA-CFs (111 ± 19 F g^–1^) and PAN-CFs (90 ± 9 F g^–1^) (Figure 7c). The excellent capacitance was attributed to the interconnected meso and micropores derived from block copolymers, offering effective routes for ion diffusion. Furthermore, 37% of the total capacitance stemmed from pseudo capacitance, according to two approaches for differentiating capacitance (Trasatti and Dunn’s methodologies) (Figure 7d). The device exhibited a 9.6 kW kg^–1^ power density at 4.5 Wh kg^–1^ energy density and an excellent extended cycle life after 10,000 cycles (Figure 7e). The Nyquist plot demonstrated that PAN-*b*-PMMA-CFs had a significantly lower charge transfer resistance (*R*_ct_) and series resistance (*R*_s_) than PAN/PMMA-CFs and PAN-CFs, indicating a substantial increase in conductivity (Figure 7f). This drastic improvement likely result from the highly interconnected mesoporous structure of PAN-*b*-PMMA-CFs with a controllable fiber diameter, reducing the transverse distance for mass transport. When optimized for axial electrical conductivity, the continuous fibers enable efficient electron transport, making them excellent electrode materials for electrochemical applications. The impacts of pore size and surface area on the electrochemical performance were also studied. The surface area positively correlates with the capacitance (Figure S1).^4^

#### 4.1.2 Batteries

**Figure 8:**
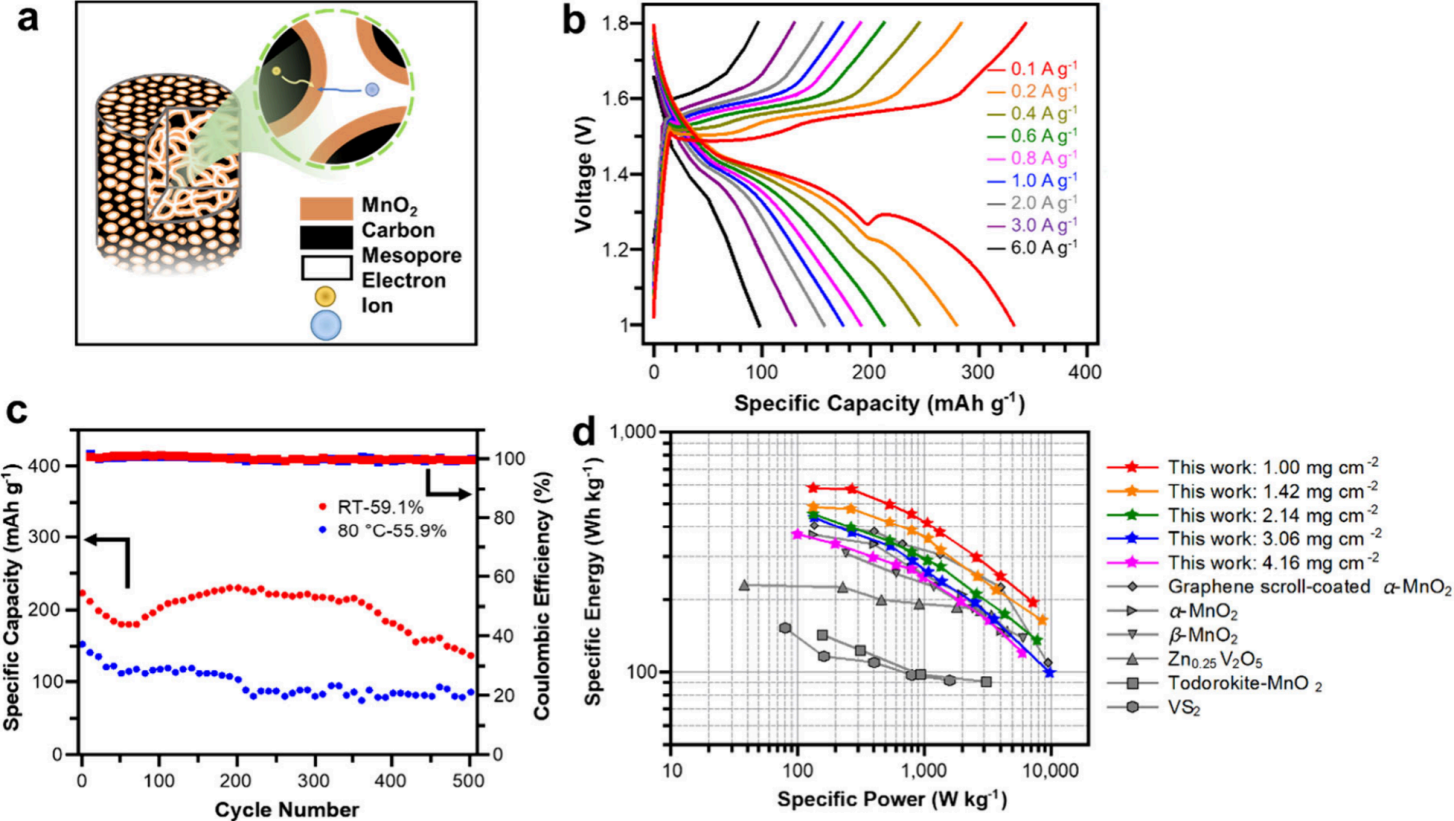
(a) Schematic of high-loading, fast-charging PCF@MnO_2_ cathode design utilizing PCFs with homogeneous mesopores, (b) Charge–discharge profiles of RT-59.1% in aqueous ZIBs, (c) Cycle stability tests of both PCF@MnO_2_ prepared at 80 °C with a MnO_2_ loading of 55.9% and prepared at RT with a MnO_2_ loading of 59.1%. (d) PCF@MnO_2_ Ragone plot compared to other cathodes. Reproduced with permission from ref (23). Copyright 2022 The Authors.

In aqueous zinc batteries, a highly relevant technology for long-duration energy storage, Zn^2+^ ions intercalate and deintercalate in the electrodes during the charge–discharge cycles.^37^ Manganese-based oxides exhibit subpar electrochemical performance due to the inherent low conductivity and significant volume changes caused by the intercalation and deintercalation process. Compounding manganese oxide with conductive PCFs is an effective way to improve conductivity and stability. Guo et al. prepared block copolymer-derived PCFs and loaded the uniform mesopores with nanoscale MnO_2_ (Figure 8a), which was tested as the cathode material in batteries.^23^ Thanks to the strong conductivity of PCF, rapid ion diffusion in nanoscale MnO_2,_ and the rapid ion transport in the fibers, the PCF@MnO_2_ electrode (MnO_2_ loading, 59.1 wt %) showed a fast charging capability, with an impressive capacity of 326 and 184 mA h g^–1^ at the current densities of 0.1 and 1.0 A g^–1^, respectively (Figure 8b). Over 500 cycles, PCF@MnO_2_ with different MnO_2_ loadings (80 °C–55.9% and room temperature (RT)–59.1%) showed stable specific capacities due to an increased tolerance to volume expansion and Mn^2+^ recovery during the cycling (Figure 8c). It is most likely the result of electric activation and Mn^2+^ additive recovery that there is a modest increase in specific capacity after 50 cycles. MnO_2_ areal loading has a significant impact on power and energy densities. Specifically, MnO_2_ loadings of 1.00 and 1.42 mg/cm^3^ in RT-incubated PCF@MnO_2_ showed improved MnO_2_-based energy and power densities due to the fast ion (de)insertion in the thin MnO_2_ layer (Figure 8d).

### 4.2 Water Purification

#### 4.2.1 Water Desalination

Desalination is a potential solution to the freshwater shortage issue because of the abundance of seawater on Earth.^38,39^ Among the many emerging methods, capacitive deionization (CDI) and solar desalination are potential alternatives to complement the existing methods for water desalination, such as reverse osmosis and thermal distillation.^38,39^

**Figure 9:**
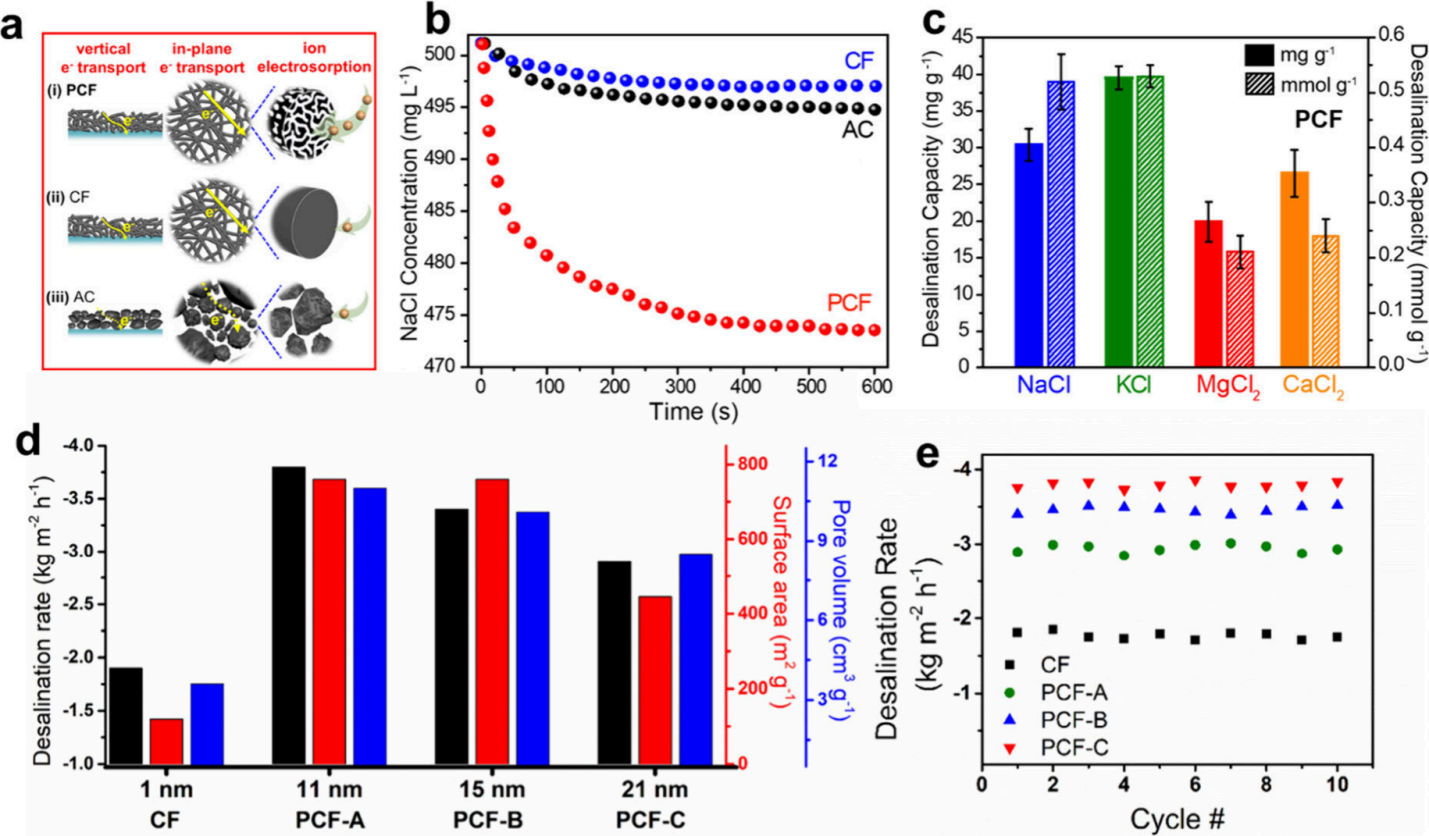
(a) Schematic of electron and ion transport in different carbon materials, including PCF, CF, and AC. (b) Concentration of NaCl during 600 s of desalination by PCF, CF, and AC in CDI cells. (c) Gravimetric and molar desalination capacities for deionization of NaCl, KCl, MgCl_2_, and CaCl_2_ deionization by PCF. Reproduced with permission from ref (41). Copyright 2020 The Authors. (d) Desalination rate, surface area, and pore volume of PCFs and CFs. (e) Desalination rate of PCFs and CFs over 10 cycles. Reproduced with permission from ref (44). Copyright 2022 American Chemical Society.

CDI removes salt ions via electrosorption^40,41^ or pseudocapacitive reactions,^42,43^ which is suitable for desalinating water with low salt concentrations. Compared with PAN-based carbon fiber (CF) and activated carbon (AC) (Figure 9a), block copolymer-based PCFs^41,44^ with high surface areas and uniformly distributed pores provided ions with continuous and effective diffusion pathways, thus improving the water desalination rate. In contrast, PAN-based CF and AC had limited mesopores and high interparticle resistance, respectively, hindering ion transport. Using symmetric electrodes adhered to Sn tape, single-cycle deionization was performed with the three types of carbon materials (i.e., AC, CF, and PCF). The salt concentration (e.g., NaCl) decreased after applying a 1.0 V bias voltage in CDI cells for 10 min (Figure 9b). PCFs achieved a Na^+^ desalination capacity of 30.4 ± 2.2 mg g^–1^, much higher than CF and AC. PCF can also remove other common cations in salt water, such as K^+^, Mg^2+^, and Ca^2+^ (Figure 9c). The highest molar desalination capacity of PCF is for K^+^ (0.53 ± 0.02 mmol g^–1^), followed by Na^+^ (0.52 ± 0.05 mmol g^–1^), Ca^2+^ (0.24 ± 0.03 mmol g^–1^), and Mg^2+^ (0.21 ± 0.03 mmol g^–1^). The molar desalination capacities for monovalent ions (Na^+^ and K^+^) were approximately double those for divalent ions (Ca^2+^ and Mg^2+^), indicating the desalination capability of PCF was mainly from electrosorption via an electric double layer.

Solar desalination converts solar energy to heat and evaporates salt water into freshwater, which is a sustainable process with minimum waste.^45,46^ The solar desalination capability of PCFs was tested using a solar simulator in a dark room. PAN-based CF and block copolymer-derived PCFs with different pore widths (21.6, 14.7, and 10.7 nm) were prepared. PCFs showed higher desalination rates than CF (Figure 9d). The highest desalination rate was ∼3.7 kg m^–2^ h^–1^ from PCFs with a pore width of 10.7 nm. The carbon fibers were robust and sustainable under continuous operation. The desalination rate of all carbon materials was stable after 10 cycles (Figure 9e). As the decrease of mesopore size, the vaporization enthalpy decreased due to the nanoconfinement effect. However, the enthalpy increased when the pore size was reduced to micropores. The interconnected porous structure in PCFs provided important factors for continuous desalination. PCFs with mesopores improved the desalination rate, and the PCFs with a mesopore size of 10.7 nm achieved the highest desalination rate.

#### 4.2.2 Organic Dye Removal

**Figure 10:**
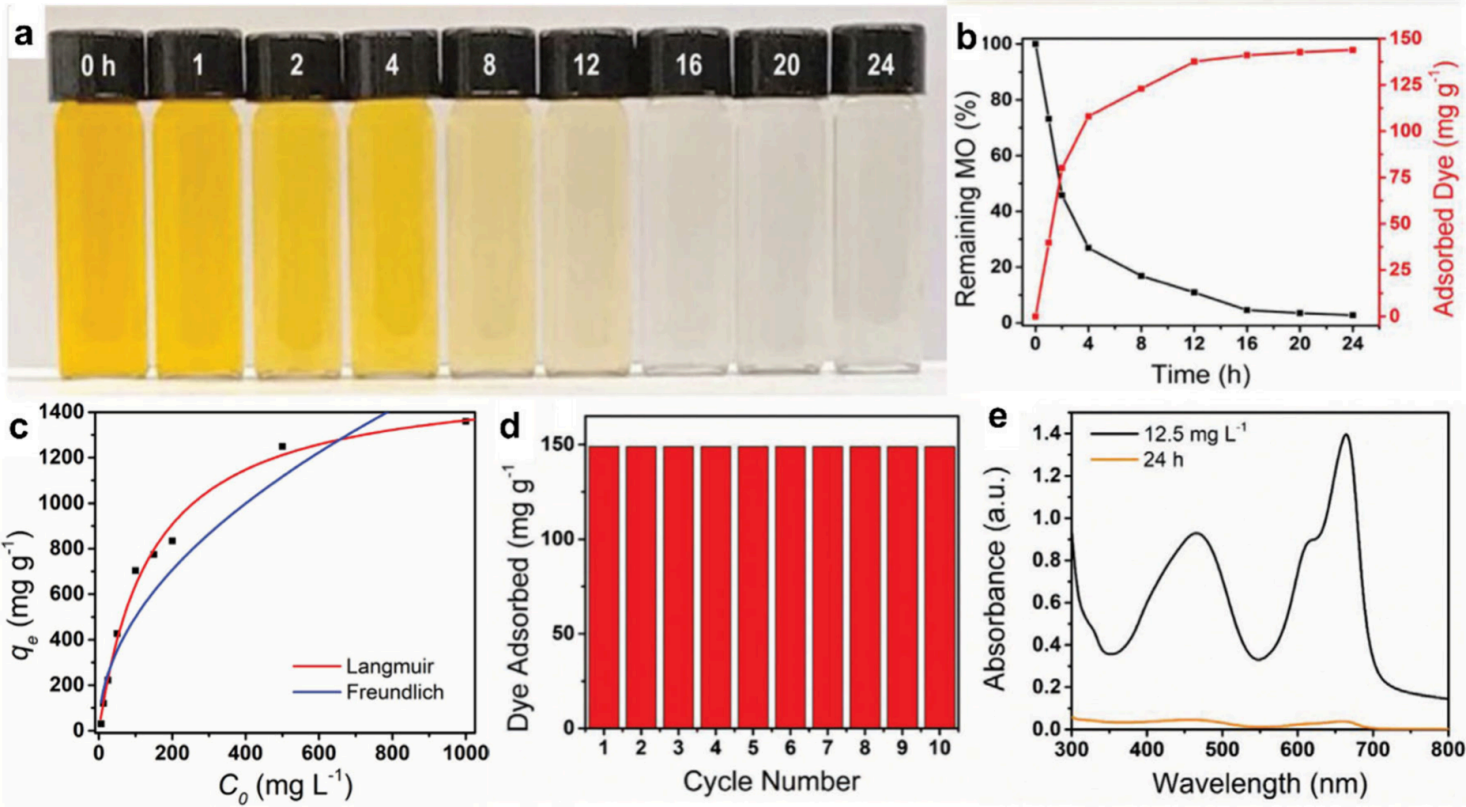
(a) Photo of MO solutions after 0–24 h of electrosorption. (b) The amount of MO adsorbed by PCF and the remaining MO% after electrosorption for 0–24 h. (c) Equilibrium adsorption of MO is fitted using the Weber-Morris and Langmuir model. (d) MO adsorption by PCF over ten cycles. (e) UV–vis spectra of MO and MB electrosorption by PCF. Reproduced with permission from ref (6). Copyright 2020 John Wiley and Sons.

The high surface area, interconnected pores, and good electrical conductivity of PCFs are also suitable for capacitive organic dye removal in wastewater.^6^ Organic dye removal is critical in the textile industry to recover dye molecules or wastewater treatment before disposal. As a proof of concept, a 0.8 V bias voltage was applied between two PCF symmetric electrodes for 24 h for the electrosorption of methyl orange (MO) (Figure 10a). The electrosorption of dye ions was rapid in the first hour, and the dye concentration in the solution dropped to 30% (Figure 10b). The electrosorption rate decreased exponentially, and the solution became almost clear after 16 h. The MO left after 24 h was <1%. The electrosorption kinetics was studied by fitting the adsorptive profiles with different models. Weber–Morris model and Langmuir model indicated that the interconnected porous structures of PCFs were beneficial for molecular diffusion into the inner sites (Figure 10c). Since the electrosorption is reversible, the adsorbed dye ions on PCFs can rearrange and desorb from the surface easily, enabling the reusability of PCFs. The adsorption loading of each cycle was close to 150 mg g^–1^ and varied <0.5% within 10 cycles (Figure 10d). PCFs could remove multiple dyes in the solution. The drastic decrease of the absorbance in ultraviolet–visible (UV–vis) spectroscopy (Figure 10e) showed the electrosorption of both MO and methyl blue (MB). Because of the opposite charges on MO and MB, the electrosorption system can be configured for selective dye removal by adjusting the cathode or anode materials.

### 4.3 Polymer Fillers and Their Role in Reinforcing Rubber Composites

Rubber and its composites are known for their mechanical properties, making them a popular choice for various consumer goods.^47−49^ Traditional fillers used to enhance the rubber properties include carbon black (CB) and silica. However, these fillers possess low dispersity and weak physicochemical interaction. The PCFs address both issues. The tunable pore size and mesoporous 3D network enhance interlocking with the polymer matrix. Moreover, the controlled content of heteroatoms (nitrogen, oxygen) mitigates filler–filler aggregation. Thus, PCFs represented a promising advancement in the field of polymer fillers.

**Figure 11:**
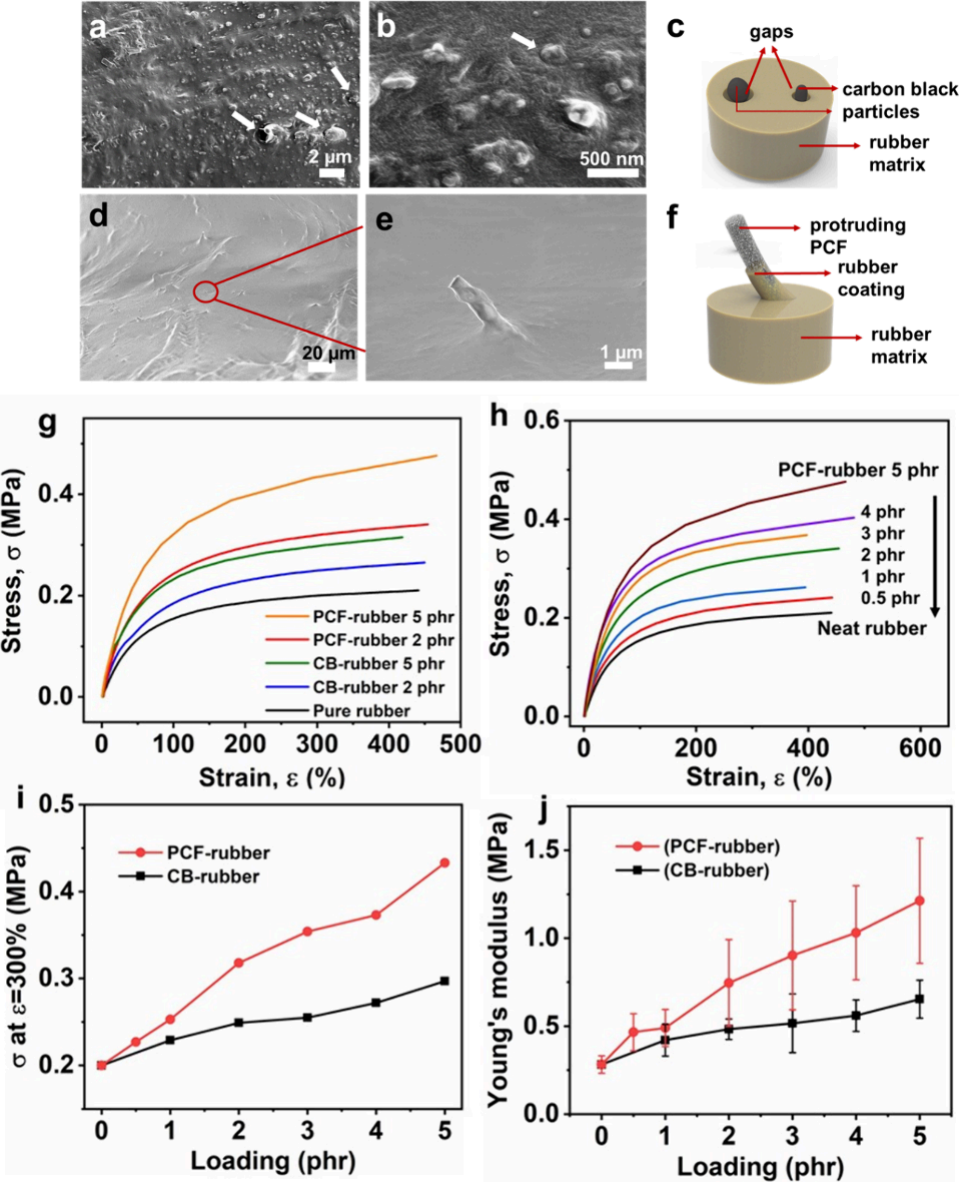
(a, b, d, e) SEM images and (c, f) scheme of CB-rubber and PCF-rubber. (g) Stress–strain curves of neat rubber, CB-rubber, and PCF-rubber at 2 and 5 phr. (h) Stress–strain curves of neat rubber and PCF-rubber at different loadings from 0.5 to 5 phr. (i) Stress at 300% strain and (j) Young’s moduli of CB-rubber and PCF-rubber at various loading of CB and PCF fillers. phr, part of filler per hundred parts of rubber. Reproduced with permission from ref (7). Copyright 2021 American Chemical Society.

In a study by Zhao et al.,^7^ PCFs were incorporated into rubber using a solution casting method. This process involved dispersing a PCF-suspension in a rubber solution, which was subsequently molded to form films. CB particles showed poor compatibility with the rubber, as evidenced by the gaps around the CB particles (Figure 11a–c). In contrast, PCFs showed excellent compatibility with the rubber, resulted in uniform mixing, exceptional wettability, and mechanical interlocking with the rubber (Figure 11d–f), which enhanced the mechanical properties. The research team experimented with various loadings of PCFs in the rubber, ranging from 0.5 to 5 parts of PCFs per 100 parts of rubber (0.5–5 phr). The results were promising, and PCF-filled rubber exhibited significantly improved storage and Young’s moduli. Dynamic Mechanical Analysis (DMA) was used to study the mechanical behavior of rubber, CB-rubber, and PCF-rubber. The key parameters considered were Young’s modulus and stress at 300% strain. Both CB and PCF additives increased the mechanical characteristics of the rubber matrix. However, notably, the strength of PCF-rubber surpassed that of CB-rubber. At a low loading of 2 parts of CB or PCF per 100 parts of rubber, the strength of PCF-rubber exceeded CB-rubber by 20.8%. Upon further increasing the loading to 5%, the strength advantage of PCF-rubber expanded to 36%. Remarkably, even at minimal loadings (0.5%), PCF-rubber exhibited significantly improved properties compared to unmodified rubber. Young’s modulus and strength of PCF-rubber were 65.4% and 19.5% higher, respectively, than those of the untreated rubber (Figure 11g–j). These findings suggest that PCF fillers are superior candidates compared to traditional CB fillers and underscore the potential of PCFs as effective fillers in composites.

## 5 Outlook and Perspectives

In this Account, we have summarized our research in developing PCFs in three aspects: (1) innovating the carbon precursors of block copolymers for PCF synthesis, (2) controlling the electrical, chemical, and mechanical properties of PCFs, and (3) demonstrating the potential of PCFs in energy storage, water purification, and polymer composites.

We examined the synthesis of PAN-*b*-PMMA via RAFT polymerization, employed electrospinning to fabricate polymer fiber mats, and applied oxidation and pyrolysis to obtain PCFs. We then discussed the critical role of different processing conditions on the properties of PCFs, including the polymer composition, pyrolysis temperature, and humidity control during electrospinning. The block copolymer composition greatly affects the domain size and microphase separation, resulting in different pore size distributions and surface areas in PCFs. The block copolymers with ϕ_PAN_ ∼ 50% offer the highest surface area thanks to the bicontinuous porous structure and interconnected pores. The pyrolysis temperature affects the decomposition of PMMA and the conversion of PAN into carbon. The humidity level during electrospinning affects the mechanical properties (e.g., flexibility) of PCFs. The nonsolvent vapor-induced effect of relatively high humidity provides a core–sheath structure of PAN and PMMA, resulting in tough and flexible PCFs with tensile strength as high as 20 MPa.

PCFs have shown high surface areas, rich functionalities, high conductivity, rapid ion transport, and good stability, and thus are excellent electrochemical electrode materials. We have applied PCFs in supercapacitors and batteries, showing a high specific capacitance and capacity. The abundant micropores and mesopores provide an effective means to fast charging and discharging. In addition, PCFs are suitable materials for water treatment including CDI, solar desalination, and organic dye removal. Results have shown that PCFs are robust and sustainable materials with high sorption capacity, better rate, and enhanced stability. The mechanical properties of PCFs have been investigated by applying them as a reinforcement filler in rubber. PCFs significantly improve the storage and Young’s moduli. Notably, PCFs can enhance the mechanical characteristics of the rubber matrix even at a minimal loading of 0.5%.

Despite the promising research on PCFs, further studies are necessary to enhance their performance and commercial viability. For improved conductivity, stability, and porosity, diverse block copolymer precursors should be investigated. A thorough examination of the correlation between PCF characteristics and processing parameters is still needed because optimal processing parameters are critical to achieving PCFs with the best mechanical and electrochemical performance. Additionally, developing scalable synthesis methods and assessing economic viability are vital for industrial deployment. There are many prospects to expand PCF applications, for instance, improved filtration systems,^50^ better biosensors for electrochemical detection of neurotransmitters,^51^ regenerative medicines,^52^ biomaterials for drug delivery,^53^ bone fracture treatment,^54^ dental implants,^55^ and biological imaging.^56^ To ensure environmental benefits, lifecycle assessment will further promote PCFs in green technologies, leveraging its unique properties to meet modern technological and environmental needs.

## Supporting Information Available

The impacts of pyrolysis temperature, pore size, and surface area of PCFs on its electrochemical performance for supercapacitor (PDF)
