# Supplementary material for: Block Copolymer Based Porous Carbon Fiber—Synthesis, Processing, and Applications
Source: Acc Mater Res. 2025 Feb 2;6(3):366–78. doi: 10.1021/accountsmr.4c00404 (PMC11959516; doi:10.1021/accountsmr.4c00404)
Supplement: Supplementary file 1 — mr4c00404_si_001.pdf [file mr4c00404_si_001.pdf]

Supporting Information of

Block Copolymer Based Porous Carbon Fiber – Synthesis, Processing, and Applications

Adeel Zia,[1] *Yue Zhang,*[1] *Akshara Paras Parekh,*[1] *Guoliang Liu*[1,2*]

1. Department of Chemistry, Virginia Tech, Blacksburg, VA 24061, USA.

2. Department of Chemical Engineering, Department of Material Science and Engineering, Macromolecules Innovation Institute, Virginia Tech, Blacksburg, VA 24061, USA.

Corresponding Author Email: gliu1@vt.edu

The impacts of pore size and surface area of PCFs on the performance of supercapacitors were studied. Serrano et al. designed a series of block copolymer precursors with different molecular weights and compositions to acquire PCFs. The electrochemical tests showed that the capacitance had a positive correlation with the surface area.[1] PCFs derived from PAN-*b*-PMMA with a molecular weight (MW) of 62-*b*-56 kDa exhibited the largest specific capacitance of 345 F g^{-1} in aqueous electrolyte with 3 M KOH at 10 mV s^{-1} **(Figure S1a).** Furthermore, all PCFs showed comparable levels of graphitization and combined series of resistance (R_s) values < 1.0 Ω **(Figure S1b)**, a feature for highly conductive carbon materials in concentrated electrolytes. Notably, an increasing mesopore width correlated to an ion diffusion resistance that drops from 0.52 to 0.22 Ω $s^{-0.5}$ and, thus, an improved rate capability.

The pyrolysis temperature of the polymer precursors strongly influences the porosity, graphitic level, heteroatom content, and electron/ion conductivity of PCFs. We have investigated the relationships between the temperature during pyrolysis (600 – 1200 °C) and the electrochemical properties of PCFs.[2] At 800 °C, PCFs exhibited the maximum gravimetric capacitance due to balanced properties such as well-defined porous structures, a notable abundance of heteroatoms, minimal charge transfer resistance, and excellent electron and ion conductivities **(Figure S1c, f)**. Asymmetric triangular patterns at 10 A g^{-1} were observed in the galvanostatic charge-discharge (GCD) curves of PCF that were pyrolyzed between 800 and 1200 °C, indicating high Coulombic efficiency due to low ion diffusion and charge transfer resistances **(Figure S1d)**. PCFs pyrolyzed at 600 °C showed the poorest rate capability due to their low electrical conductivity **(Figure S1e, f)**. However, when the pyrolysis temperature was raised to 1200 °C, the PCFs excelled in rate capability due to superior electrical conductivity, large pore volume, and minimal diffusion resistance **(Figure S1e)**.

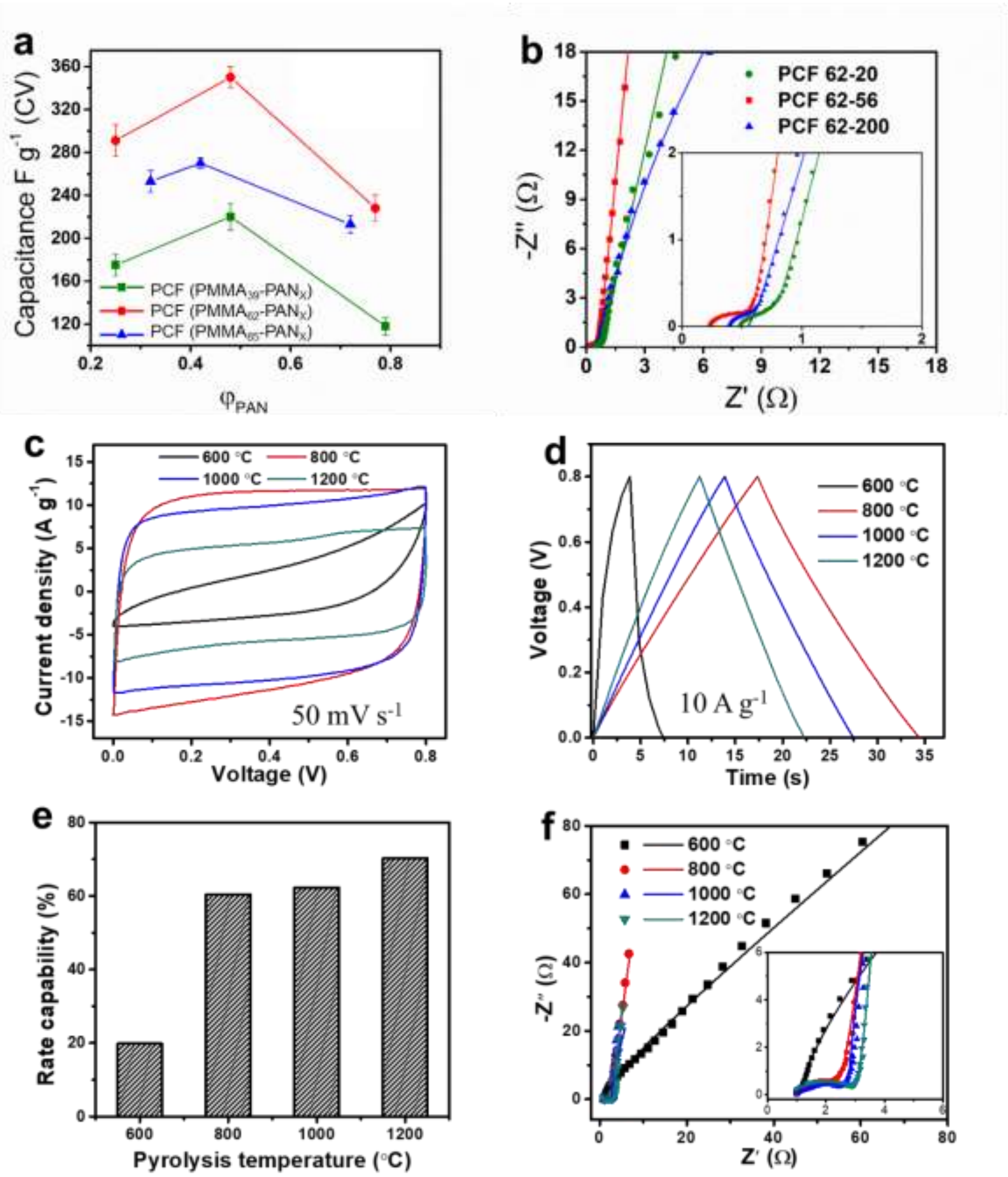

Figure S1. (a) Average gravimetric capacitances in 3 M KOH of PCFs (derived from different MW of PAN-*b*-PMMA) at 10 mV s^{-1}, (b) Nyquist plots. Reproduced with permission from reference.[1] Copyright 2019 American Chemical Society. (c) CV of PCF after pyrolysis at different temperatures with a scan rate of 50 mV s^{-1}, (d) GCD curves at a current density of 10 A g^{-1}, (e) Rate performance of the PCFs at different pyrolysis temperatures, and (f) Nyquist graphs with a perturbation of 10 mV are produced in the frequency

range of 100 kHz to 0.1 Hz. Reproduced with permission from reference.[2] Copyright 2020 The Royal Society of Chemistry.

References

1. Serrano, J. M.; Liu, T.; Khan, A. U.; Botset, B.; Stovall, B. J.; Xu, Z.; Guo, D.; Cao, K.; Hao, X.; Cheng, S.; Liu, G. Composition Design of Block Copolymers for Porous Carbon Fibers. *Chem. Mater.* **2019**, *31* (21), 8898-8907.
2. Zhou, Z.; Liu, T.; Khan, A. U.; Liu, G. Controlling the Physical and Electrochemical Properties of Block Copolymer-Based Porous Carbon Fibers by Pyrolysis Temperature. *Mol. Syst. Des. Eng.* **2020**, *5* (1), 153-165.
